# Experimental evolution reveals that sperm competition intensity selects for longer, more costly sperm

**DOI:** 10.1002/evl3.13

**Published:** 2017-06-07

**Authors:** Joanne L. Godwin, Ramakrishnan Vasudeva, Łukasz Michalczyk, Oliver Y. Martin, Alyson J. Lumley, Tracey Chapman, Matthew J. G. Gage

**Affiliations:** ^1^ School of Biological Sciences University of East Anglia Norwich Research Park Norwich NR4 7TJ United Kingdom; ^2^ Institute of Zoology Jagiellonian University Kraków Poland; ^3^ ETH Zürich Institute of Integrative Biology Zürich Switzerland

**Keywords:** Anisogamy, directional selection, sexual selection, stabilising selection, Tribolium

## Abstract

It is the differences between sperm and eggs that fundamentally underpin the differences between the sexes within reproduction. For males, it is theorized that widespread sperm competition leads to selection for investment in sperm numbers, achieved by minimizing sperm size within limited resources for spermatogenesis in the testis. Here, we empirically examine how sperm competition shapes sperm size, after more than 77 generations of experimental selection of replicate lines under either high or low sperm competition intensities in the promiscuous flour beetle *Tribolium castaneum*. After this experimental evolution, populations had diverged significantly in their sperm competitiveness, with sperm in ejaculates from males evolving under high sperm competition intensities gaining 20% greater paternity than sperm in ejaculates from males that had evolved under low sperm competition intensity. Males did not change their relative investment into sperm production following this experimental evolution, showing no difference in testis sizes between high and low intensity regimes. However, the more competitive males from high sperm competition intensity regimes had evolved significantly longer sperm and, across six independently selected lines, there was a significant association between the degree of divergence in sperm length and average sperm competitiveness. To determine whether such sperm elongation is costly, we used dietary restriction experiments, and revealed that protein‐restricted males produced significantly shorter sperm. Our findings therefore demonstrate that sperm competition intensity can exert positive directional selection on sperm size, despite this being a costly reproductive trait.

**Impact Summary**

When sexual reproduction evolved, it is theorized that a phenomenon known as “disruptive selection” drove the evolution of gametes into two different types ( = anisogamy), making them equally adapted in their own ways for reproduction. Eggs were primarily shaped by the requirement to resource the embryo, and therefore needed to be large (making them few in number). Sperm was primarily shaped by the requirement to locate and fertilize eggs and, when there was competition for fertilizations, needed to be plentiful (making them small in size). For sperm, therefore, widespread numerical competition for fertilizations is thought to have made them numerous and tiny. We now know, however, that sperm are extremely diverse in size and shape, so their evolution may be more complicated than a simple drive to maximize numbers. To better understand these important cells, we used laboratory evolution with an insect model to measure how competition shapes sperm size. We maintained lines of beetles for over five years under identical conditions except, at every adult generation, we created mating regimes presenting very high or very low intensities of sperm competition. After ∼80 generations, we found that sperm competitiveness had diverged significantly: sperm of males from the high competition regime achieved 20% higher fertilization success in competition than sperm of males evolving under the low competition regime. Importantly, we also found that these more competitive males had evolved significantly longer sperm, indicating that sperm competition can select for qualitative aspects of sperm form and function, and that competition is not just a numbers game. To assess whether sperm elongation places demands on males, we used dietary restriction experiments and found obvious costs to producing longer sperm because protein‐restricted males developed shorter sperm. Our results demonstrate that competition in the struggle to reproduce can increase sperm size, despite this carrying costs, and therefore that the selective forces controlling the evolution of many, tiny sperm are more complex than originally assumed.

## Introduction

Our understanding of the evolution of sperm form and function has its roots in anisogamy theory, where numerical competition for fertilizations was proposed to shape the fundamental phenotype of a male gamete that was produced in vast numbers, achieved via minimizing sperm size and increasing testicular investment (Parker et al. 1972, 1997; Parker 1982; Lessells et al. 2009; Parker and Pizzari 2010). It was this logic that answered the question “why are there so many tiny sperm?” (Parker et al. 1972; Parker 1982; Pizzari and Parker 2009). There is good evidence that numerical superiority of sperm (the “raffle principle”) is indeed shaped by sperm competition (e.g., Wedell et al. 2002; Parker and Pizzari 2010; Kelly and Jennions 2011), and we also recognize that varying selection from sperm competition is a near‐universal force among sexual reproducers (Taylor et al. 2014). However, we also recognize that sperm cells are not always minimally sized for the production of maximal numbers. In fact, spermatozoa are the most morphologically diverse eukaryotic cell types known (Pitnick et al. 2009a); even sperm size alone varies more than 8000‐fold, from the diminutive gametes of the male braconid parasitoid wasp *Cotesia congregata* (7 μm; Uzbekov et al. 2017) to the giant sperm of *Drosophila bifurca* (58,290 μm; Pitnick et al. 1995). Most of this profound variation remains unexplained, but the diversity in form and function suggests that the selective forces shaping sperm form and function are more complex than a basic drive to win fertilization competitions by maximizing sperm number and minimizing sperm size.

There is increasing attention to the possibility that much of the huge variation in spermatozoa could be the result of postcopulatory sexual selection, when the forces of competition and choice act on gamete biodiversity (Snook 2005; Rowe et al. 2015; Lüpold et al. 2016). We now know that fertilization outcome can depend on variation in form, function, and identity of competing sperm (Pizzari and Parker 2009), and that females have evolved mechanisms at the cryptic level of the gamete to control fertilization and influence paternity (Pitnick et al. 2009b). The evolution of spermatozoa will therefore be subject to focused selection from a complexity of interacting forces arising both from cryptic female choice, and competition between rival males and their sperm. Previously, results from cross‐species studies were mainly used to infer how postcopulatory sexual selection had shaped sperm cell phenotypes, with evidence that increasing sperm competition intensity can have positive, negative, or neutral effects on sperm size variation (reviewed in Snook 2005). Findings have therefore been mixed, with one possible reason being due to differences between species in the additional impact of female‐controlled influences on sperm competition, cryptic choice, and fertilization. By analyzing both sperm number and sperm size simultaneously, for example, in the context of varying selection from female size and tract dilution, Lüpold and Fitzpatrick (2015) showed that there is positive selection from sperm competition intensity on both sperm number and sperm size across mammals. However, responses to selection were greater for sperm number, and greater still in those larger species where sperm dilution in the competitive arena of the female tract was more likely to exist (Lüpold et al. 2016). Comparative studies can also be sensitive to assumptions about phylogenetic relatedness. Previous analyses of the relationship between sperm competition intensity and sperm size across mammals have found both positive and neutral relationships, which could result from the use of different phylogenies (Gomendio and Roldan 1991; Gage and Freckleton 2003; Tourmente et al. 2011), and analyses across passerine birds have revealed significant relationships between sperm competition and sperm dimensions, but these differed depending in Family, being positive in the Fringillidae, and negative in the Sylviidae (Immler and Birkhead 2007).

Here, we use long‐term experimental evolution within a single species to measure how sperm length evolves across independently replicated lineages. The flour beetle, *Tribolium castaneum*, is promiscuous and females store sperm (Fedina and Lewis 2008; Michalczyk et al. 2010; Michalczyk et al. 2011; Lumley et al. 2015). Sperm form and function are therefore expected to be key targets of postmating sexual selection. Manipulation of adult sex ratios was used to create Male‐biased (90 males to 10 females) intense sperm competition populations, contrasting with Female‐biased (10 males to 90 females) relaxed sperm competition populations. After 77–83 generations of this experimental evolution, we measured how ejaculate competitiveness, overall male investment to spermatogenesis, and sperm size had evolved. We hypothesized that, if sperm competitiveness responds to divergent intensities of sperm competition, the “raffle principle” within anisogamy theory (Parker et al. 1972; Pizzari and Parker 2009) should drive sperm size to either decrease, or remain at some biological minimum within investment to spermatogenesis in the testis, maximizing numerical superiority of sperm within competitions for fertilizations. On the other hand, if competition selects for qualitative adaptations in sperm for winning fertilizations (Lüpold et al. 2016), elevated postcopulatory sexual selection could increase sperm size. Additionally, to test the hypothesis that any evolution of sperm length is constrained by the existence of costs, we also employed dietary restriction experiments that limited the amount of resource available for spermatogenesis, and then assessed the relative impact upon sperm elongation. Therefore, as well as measuring how experimental variation in postcopulatory sexual selection shapes sperm competitiveness and sperm length evolution, this study also empirically examines the costs of sperm elongation for males.

## Methods

### EXPERIMENTAL EVOLUTION UNDER DIVERGENT SPERM COMPETITION INTENSITIES

Was conducted with beetles of the widely used Georgia 1 (GA1) “wild‐type” strain, originating from the Beeman Lab (United States Department of Agriculture). Populations were maintained at standard conditions of 30°C and 60% humidity, within ad libitum medium consisting of 90% organic white flour, 10% brewer's yeast, and a thin layer of oats to aid traction. Divergent operational sex ratios during the adult life stage were used to apply a Male‐biased (90 males to 10 females), intense sperm competition regime, contrasted against a Female‐biased (10 males to 90 females), relaxed sperm competition regime. Male and female mating potential and promiscuity assays demonstrate that our male‐ versus Female‐biased regimes create extreme divergence in sperm competition intensities; full details are in Michalczyk et al. (2011) and Lumley et al. (2015). The regimes were structured so that theoretical effective population size (Wright 1931) was equalized to minimize the opportunity for differences in genetic drift and/or inbreeding to influence either the male‐ or Female‐biased regimes. By structuring our adult regimes as either 90:10 or 10:90, we generated adult *N_e_* throughout of 36; post hoc genetic testing using a suite of microsatellites confirmed that we had retained equal levels of heterozygosity between the regimes (Lumley et al. 2015). Three independent lines within each regime were maintained. For each independent line, at every generation, pupae were sexed to ensure virginity. Adults were then placed in fresh medium for seven days for mating, sperm competition, and oviposition, after which they were removed and eggs/larvae left to develop in standardized conditions until pupae were ready for beginning the next generation.

### SPERM COMPETITIVENESS

**Figure 1:**
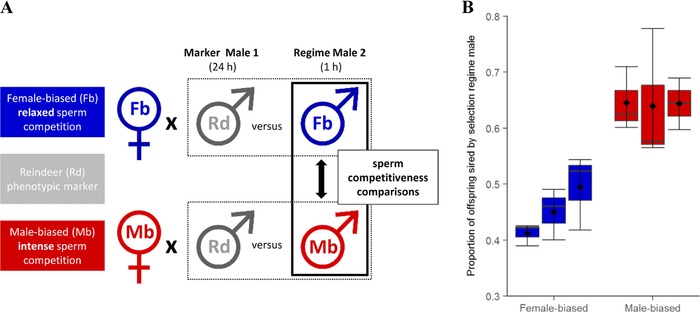
Experimental design and outcome of a sperm competition assay comparing ability of sperm from relaxed Female‐biased (Fb), and intense Male‐biased (Mb), sperm competition regime males to compete for fertilizations against sperm from marker Reindeer (Rd) males. (A) Experimental design of sperm competition assay. (B) Proportion of offspring sired by males from contrasting Female‐biased and Male‐biased sperm competition regimes, following sperm competition through 20 days of oviposition. Male‐biased males sired a significantly greater proportion of offspring (negative binomial GLMM: $\chi_{\left(1\right)}^{2}$= 5.58, *P* = 0.02). Data grouped by independent line (*n* = 13–17 replicates × 3 crosses per line; average number of offspring scored across *n* = 282 sperm competitions = 158 (± 3 SEM), range 24–295).

Figure 1 of ejaculates from individual males evolved under contrasting sperm competition intensity regimes was assessed following 77 generations of experimental evolution. Competition took place between an experimental male and a marker mutant “Reindeer” (Rd) male, allowing paternity to be assigned (Lewis et al. 2005; Tregenza et al. 2009; Fig. 1A). The Rd mutation is dominant and homozygous within the Rd population, therefore all offspring sired by a Rd male have distinctive swollen antennae, while offspring of the sex ratio treatment regime males have wild‐type (WT), filiform antennae. All adults were virgin and 10–12 days posteclosion when used, and experimentally evolved males were isolated at the pupal stage to equalize any developmental effects. Females were paired with an Rd male for 24 h in a 1 cm diameter 7 mL vial containing 2 g fresh medium, allowing frequent mate encounter and ample opportunities for mating and full sperm storage. In *T. castaneum*, even a single mating is sufficient for females to fertilize 700 eggs across four months of oviposition (Bloch Qazi et al. 1996), and both males and females mate frequently (Fedina and Lewis 2008). We can therefore assume that virgin Rd males paired for 24 h with single virgin females in these conditions will fill the limited spermathecal and bursal storage sites with Rd sperm (Bloch Qazi et al. 1996), so that the subsequent competitor males must win fertilizations within females that have been fully inseminated. After 24 h, Rd males were removed and females were presented with an unmated experimental focal male from either the male‐ or Female‐biased sperm competition intensity regime, pairing Male‐biased males with Male‐biased females, and vice versa. Pairs were left in an empty 1 cm diameter 7 mL vial to mate for 1 h, after which males were removed. After the second mating, females were transferred to Petri dishes of fresh medium, and left to oviposit across two 10‐day blocks before being removed. Thus, the outcomes of sperm competitions lasting 20 days were recorded, which typically accounts for ∼50% of the total offspring production by females from such a mating period before they run out of functional sperm (M. J. G. Gage, unpubl. data). Eggs/larvae were left to develop in individual Petri dishes, with the number of each phenotype (Rd and wild type) counted as adults. Females from the respective experimental evolution regime were used in sperm competition experiments to maintain any coevolved male–female effects within regimes, and to avoid the possibility for differential female effects when paired with males of different experimental evolution populations. To balance within‐line versus between‐line coevolutionary effects, males were competed with females from their own line and with females from the other two independent replicate lines within the regime, applying a balanced design to equalize within‐ versus between‐line effects (Fig. S1). There was no evidence of any within‐ versus between‐line influences on differential fertilization success, under either intense or relaxed evolutionary histories of sperm competition (Fig. S2).

### SPERM LENGTH FOLLOWING EXPERIMENTAL EVOLUTION

**Figure 2:**
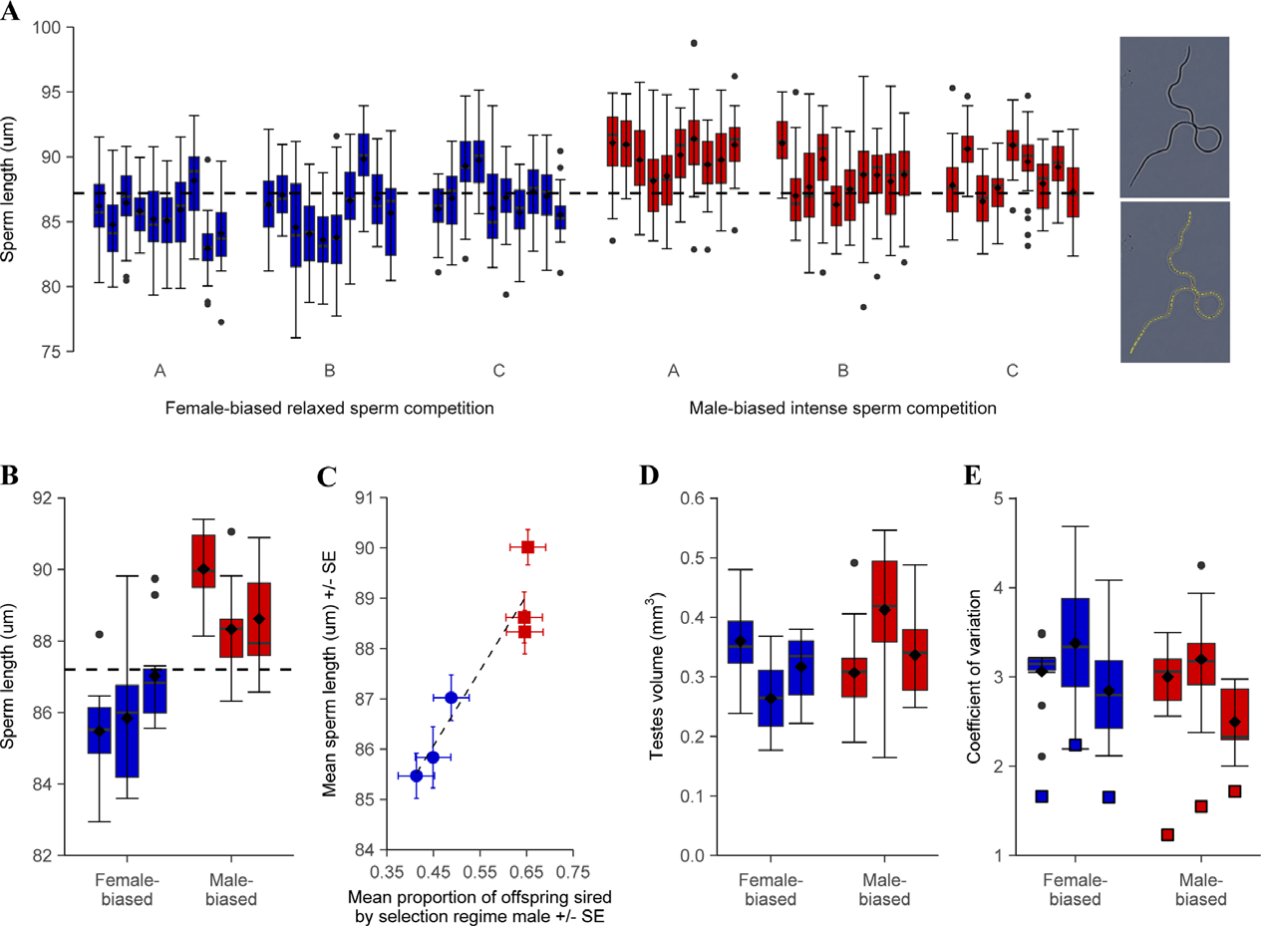
Sperm length and variance of males from relaxed Female‐biased (blue) versus intense Male‐biased (red) sperm competition histories. (A) Sperm length per male (*n* = 30 sperm × 9–10 males × 3 lines = 870–900 total sperm in either treatment), and sperm micrograph and measuring technique using "ImageJ" image analysis package (see Methods). The difference between regimes was highly significant (LMM $\chi_{\left(1\right)}^{2}$ = 6.69, *P* < 0.01). Dashed line shows mean sperm length of ancestral GA1 males (86.8 μm) to compare increases and decreases within either selection regime. (B) Sperm length grouped by independent line and dashed line for ancestral mean. (C) Correlation between sperm length and sperm competitive ability across Female‐biased (blue circles), and Male‐biased (red squares) independent lines (*r* = 0.94). (D) Testes volume (*n* = 15 males per population) also did not differ between regimes (LMM: $\chi_{\left(1\right)}^{2}$= 1.13, *P* = 0.29). (E) Within‐ and between‐male coefficients of variation (CVs) in sperm length. Neither within‐male CVs (boxplots; *n* = 30 sperm × 9–10 males per population), nor between‐male CV (filled square markers) calculated using mean sperm length of each male (*n* = 9–10 males per population) differed significantly between regimes (within; LMM $\chi_{\left(1\right)}^{2}$= 0.79, *P* = 0.37, between; *W* = 7, *P* = 0.40).

Was measured in the same replicate lines and regimes after 83 generations of experimental evolution, using similar 10‐day to 12‐day posteclosion unmated males, sexed and isolated at the pupal stage. Sperm were recovered using microdissection from females, soon after mating and spermatophore deposition. Pairs of beetles (*n* = 9–10 males × 3 independent populations = 29–30 total males per sex ratio regime treatment) were placed in 1 × 1 cm^2^ mating arenas at standard conditions for 30 min to allow spermatophore transfer. Females were then removed and decapitated, and the reproductive tract isolated by extruding the ovipositor and gently pulling to detach it from the abdomen. This was placed in a drop of buffer (0.9% NaCl) and the spermatophore isolated. Finally, the spermatophore was transferred to a 10 μL drop of fresh buffer on a clean microscope slide, teased open, and the slide flooded with further buffer to disperse the sperm. Slides were left to dry, then dipped in distilled water to remove buffer salt residue, and redried. Cleared slides were viewed with phase contrast, at 60× magnification and images were captured. Total sperm length was measured by creating a segmented line that traced the entire length of the cell using the “ImageJ” image analysis package (Schneider et al. 2012; inset Fig. 2A). Thirty sperm per male were measured (*n* = 30 sperm × 9–10 males × 3 lines = 870–900 total sperm in either treatment). Sperm were measured by two investigators. To assess repeatability, 20 sperm were measured by both investigators, and the intraclass correlation coefficient (ICC) calculated (Lessells and Boag 1987; Nakagawa and Schielzeth 2010). Repeatability was found to be very high, ICC = 0.99 (± 0.005), with close correlations between pairs of measurements (*r* = 0.99), and a Bland‐Altman plot revealed consistent agreement between the investigators across the parameter range (Bland and Altman 1986; Bartlett and Frost 2008).

### TESTIS SIZE VARIATION FOLLOWING EXPERIMENTAL EVOLUTION

Was measured in the same replicate lines after 100/107 generations. Ten‐day to 12‐day posteclosion unmated males, sexed and isolated at the pupal stage, were frozen and elytra length (*n* = 10 males × 3 independent populations = 30 males per sperm competition regime) and testes volume (*n* = 15 males × 3 independent populations = 45 males per sperm competition regime) were measured. Testes were dissected out and images of the follicles, which make up the testes in *T. castaneum*, were captured. “ImageJ” (Schneider et al. 2012) was then used to measure the circumference of the follicle, and volume calculated by assuming a spherical shape. Where possible, all 12 follicles per male were measured, however, where this was not possible due to damage to fragile follicles during dissection, total testes volume was calculated as mean measured follicle size multiplied by 12. To assess the accuracy of this estimate, uniformity of follicle size was investigated by calculating the ICC of two randomly selected follicles per male. Uniformity was found to be very high (ICC = 0.99). Hence, our measure gave an accurate estimate of total testis volume. Mean number of measured follicles was 8.82 (± 0.22) per male.

### DIETARY RESTRICTION EFFECTS ON BODY SIZE, TESTES VOLUME, AND SPERM LENGTHS

Were measured in *T. castaneum* males taken from a standard stock population of Krakow super strain (KSS) created by Ł. Michalczyk in 2008. Eggs were collected and randomly assigned to either control medium (90% organic white flour and 10% brewer's yeast) or a protein‐restricted medium in which brewer's yeast was not added (0% yeast). Ten‐day to 12‐day posteclosion unmated males, sexed and isolated at the pupal stage, were frozen and elytra length and testes volume (*n* = 32 males per nutritional treatment), and sperm length (*n* = 15 sperm x 32 males per nutritional treatment) were measured as previously described.

### STATISTICAL ANALYSES

Were conducted in “R” (R Core Team 2015), with “plyr” (Wickham 2015), “pastecs” (Grosjean et al. 2014), “car” (Fox et al. 2015), and “stats” (R Core Team 2015) packages used for data exploration, descriptive statistics, and testing assumptions. Figures were created using “ggplot2” (Wickham and Chang 2015). For all boxplots, a horizontal line indicates the median, boxes indicate the interquartile range (IQR), whiskers indicate points within 1.5 IQR, and any data not included in the box and whiskers are shown as outliers (small filled points). An additional point (filled diamond) was added to display the mean. Linear and generalized linear mixed effects models (LMM and GLMM, respectively), with appropriate error distributions, were used to include random effects to account for nesting in the data. Mixed models were fitted by maximum‐likelihood and likelihood ratio tests and AIC values were used to compare models with and without the factor of interest (Crawley 2013). All models were implemented in “lme4” (Bates et al. 2015) unless otherwise stated.

*Sperm competitiveness*, measured as the proportion of offspring sired by males from experimentally evolved regimes, was compared by constructing a GLMM, with a negative binomial error structure to account for overdispersion in the data, using the “glmmADMB” (Fournier et al. 2012) and “R2admb” (Bolker et al. 2015). The response variable was entered as a paired variable containing the number of offspring sired by each male to retain information on sample size within the model (Crawley 2013). A maximal model was fitted with “sperm competition intensity” (Female‐ or Male‐biased) and "cross" (within or between regime replicate) entered as fixed effects. Cross was then dropped from the fixed effects as it did not significantly improve the explanatory power of the model. Female line (A, B, C), nested within male line (A, B, C), were entered as random effects to check for any within‐ versus between‐line compatibility, and account for nesting in the data. To check for differential offspring mortality effects, total offspring production was also compared between female‐ and Male‐biased regimes by constructing a LMM with the same fixed and random effects structure.

*Total sperm length* was compared between experimental evolution regimes using an LMM with sperm competition intensity regime (female‐ or Male‐biased) entered as a fixed effect and replicate male (a–j), nested within replicate line (A, B, C) as random effects. In addition, a Spearman correlation, carried out using the “Hmisc” package (Harrell et al. 2016), was used to assess the association between mean sperm length and mean sperm competitiveness across treatments (*n* = 6).

*Sperm length variance* was calculated as a standardized coefficient of variation (CV) both within males (CV_wm_) and between males (CV_bm_) for each independent treatment replicate. The within male CV for a population is a mean of 10 individual male CVs. The between‐male CV for a population was calculated using the mean sperm length of each male. A linear mixed effects model was fitted to compare within male CV (*n* = 9–10 × 3 lines = 29–30 total CV_wm_ per treatment) between regimes. A maximal model was fitted with experimental evolution regimes (female‐ or Male‐biased) entered as the fixed effect, and line (A, B, C) entered as a random effect. An unpaired Wilcoxon rank‐sum test was used to compare between male CVs (*n* = 1 × 3 populations per treatment).

*Testes size and elytra length* were compared between sexual selection regimes using LMMs with sperm competition intensity regime (female‐ or Male‐biased) entered as a fixed effect and replicate line (A, B, C) entered as random effects.

*Male morphometric and reproductive traits* were compared between control and protein‐restricted dietary treatments using *t* tests (absolute testes volume and within male sperm length variance [CV]) or the nonparametric unpaired Wilcoxon rank‐sum test (elytra length). In addition, relative testes volume was compared between dietary treatments using analysis of covariance (ANCOVA) with elytra length incorporated as a covariate to account for allometry in the growth of body parts. Finally, a linear mixed model was used to compare sperm length, with diet as a fixed effect, and male (1 to 32) as a random effect to account for nesting of the data.

## Results

### SPERM COMPETITIVENESS

After 77 generations of experimental evolution under either intense or relaxed competition for fertilizations, sperm competitiveness had significantly diverged between regimes. Sperm from Male‐biased males won significantly greater numbers of fertilizations across 20 days of competition and oviposition than did sperm from Female‐biased males (negative binomial GLMM: $\chi_{\left(1\right)}^{2}$ = 5.58, *P* = 0.02; Fig. 1B). Scoring an average of 158 (± 3 SEM) offspring per competition, and with regime males mated second, Male‐biased, intense sperm competition regime males won 20% more fertilizations (65% ± 4 SEM, *n* = 143 competitions) than males from the Female‐biased, relaxed sperm competition regime (45% ± 4 SEM, *n* = 139 sperm competitions). Removing those competitions where either of the males gained zero or 100% paternity (*n* = 12 of 143 Male‐biased competitions, and 13 of 139 Female‐biased competitions) to control for the possibility that failed matings explained the paternity biases, did not change the results, with paternity share still showing a 17% difference between regimes (negative binomial GLMM: $\chi_{\left(1\right)}^{2}$ = 4.72, *P* < 0.05). Likewise, there was no indication that differential offspring mortality explained the male‐ versus Female‐biased paternity differences, because there was no significant difference between the regime crosses in the numbers of adult offspring that were produced (LMM: $\chi_{\left(1\right)}^{2}$ = 2.95, *P* = 0.09). Moreover, the direction of any difference was conservative to the difference in paternity, with Male‐biased regime sperm competitions (where more offspring were sired by the experimental evolution males) yielding slightly fewer total offspring (*n* = 151 ± 8 SEM) than the Female‐biased trials (166 ± 6 SEM).

### SPERM LENGTH

In addition to superior competitive ability, sperm produced by males from the Male‐biased, intense competition regime were significantly longer than those of males derived from the Female‐biased, relaxed competition regime (LMM: $\chi_{\left(1\right)}^{2}$ = 6.69, *P* < 0.01; Fig. 2A and B). Mean (± SEM) sperm length of Male‐biased males was 89.0 μm (± 0.57) compared to 86.1 μm (± 0.40) in Female‐biased males. Comparisons of the experimentally evolved sperm length distributions against their ancestral stock population revealed that average sperm length had both increased and decreased in the high and low sperm competition regimes, respectively (see Fig. 2A and B). In addition, there was a significant positive association across the six independent selection lines between average sperm length and mean competitive ability (Spearman correlation: *r* = 0.94, *n* = 6, *P* < 0.01; Fig. 2C).

### SPERM LENGTH VARIANCE

In addition to evidence for directional selection, we explored whether sperm competition intensity had also exerted stabilizing selection so that sperm length had evolved to a narrower optimum by comparing CVs between regimes (Lifjeld et al. 2010). Despite evidence for significant divergence in sperm competitiveness and sperm length, we found that neither within‐male CVs (LMM: $\chi_{\left(1\right)}^{2}$ = 0.79, *P* = 0.37), nor between‐male CVs (Wilcoxon rank sum test: *W* = 7, *P* = 0.4) differed between sperm competition selection regimes (Fig. 2E).

### TESTES SIZE AND BODY SIZE

Relative investment into spermatogenesis was similar for males from both male‐ and Female‐biased regimes, with no differences in testes volume (LMM: $\chi_{\left(1\right)}^{2}$ = 1.13, *P* = 0.29; Fig. 2D) or elytra length (LMM: $\chi_{\left(1\right)}^{2}$ = 0.40, *P* = 0.53) between males from contrasting regimes.

### DIETARY RESTRICTION AND SPERM LENGTH

**Figure 3:**
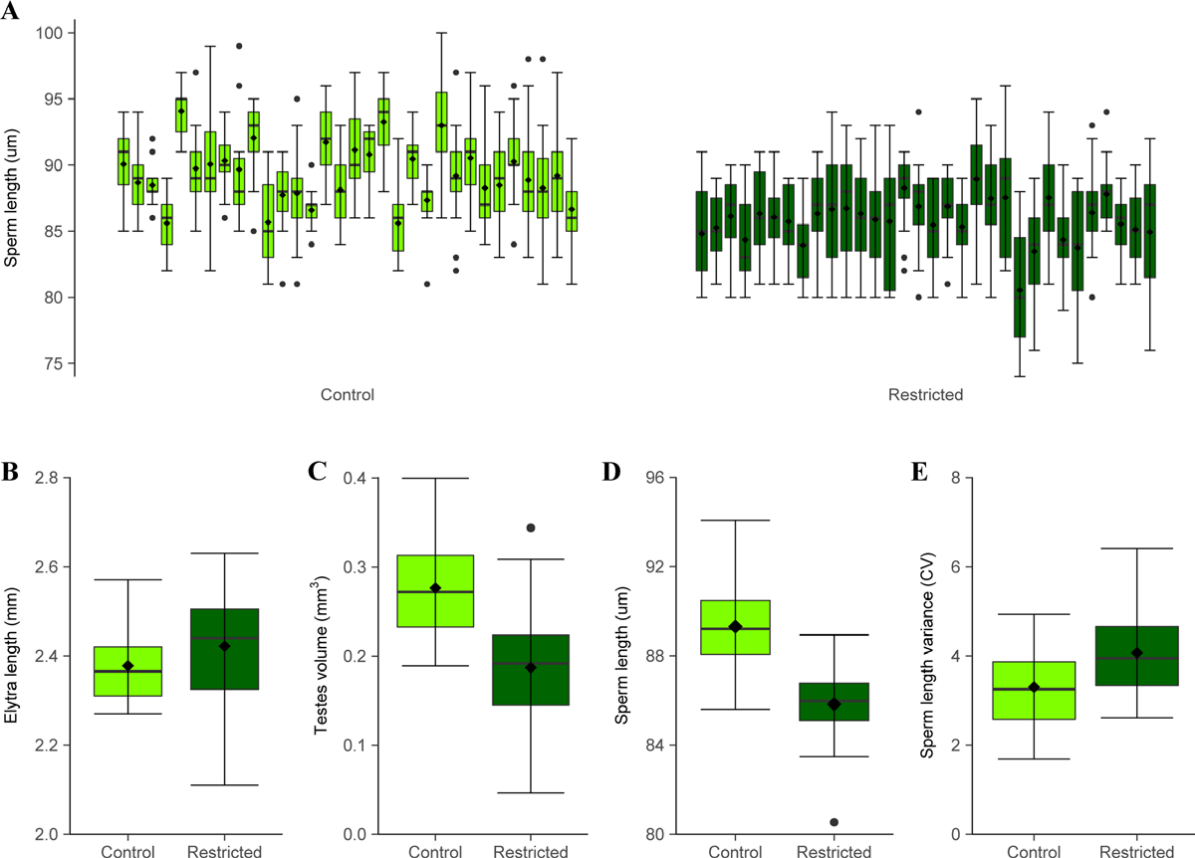
Comparison of morphometric and reproductive traits in males reared and maintained under control (light green) versus protein‐restricted (dark green) diets (*n* = 32 males per dietary treatment). (A) Sperm length per male (*n* = 15 sperm per male). (B) Elytra length did not differ between diets (Wilcoxon rank sum test: *W* = 383, *P* = 0.08). (C) Testes volume was significantly greater in control males (absolute testes volume; *t*_62_ = 5.74, *P* < 0.01, relative testes volume; ANCOVA *F*_1_,_61_ = 33.58, *P* < 0.01). (D) Mean sperm length (*n* = 15 sperm × 32 males = 480 total sperm in either treatment) was significantly longer in control males (LMM: $\chi_{\left(1\right)}^{2}$= 204.09, *P* <0.01). (E) Within‐male coefficient of variation (CV) in sperm length was significantly higher in protein‐restricted males (*t*_62_ = −3.40, *P* < 0.01).

Males reared and maintained under protein‐restricted conditions without supplementary yeast showed reduced investment in spermatogenesis, with significantly smaller absolute (*t*_62_ = 5.74, *P* < 0.01) and relative (ANCOVA: *F*_1_,_61_ = 33.58, *P* < 0.01) testis sizes than controls (Fig. 3C). Importantly, these males suffering dietary constraints to spermatogenic investment produced sperm cells that were significantly shorter than those from males reared on a standard 10% yeast diet (LMM: $\chi_{\left(1\right)}^{2}$ = 204.09, *P* <0.01; Fig. 3A and D). Sperm size variance was also greater within males suffering dietary restriction (*t*_62_ = ‐3.40, *P* < 0.01), supporting the idea that environmental stress reduced male ability to produce more uniform, as well as more elongate, sperm cell phenotypes (Fig. 3E).

## Discussion

After more than six years of experimental evolution under controlled but widely differing intensities of sexual selection and sperm competition (Lumley et al. 2015), we found replicated evidence for significant divergence in both sperm competitiveness and sperm size. In competition with sperm from standardized marker males, ejaculates from intense Male‐biased selection histories won an average of 65% of the fertilizations, whereas sperm from relaxed Female‐biased selection histories only achieved 45% fertilization success (Fig. 1B). In parallel with the divergence in sperm competitiveness, we also found significant differences in sperm length under strong versus weak selection from sperm competition, and both increases and decreases relative to the ancestral population average (Fig. 2A and B). Contrary to basic expectations from the “raffle principle” (Parker et al. 1972; Parker 1982; Pizzari and Parker 2009), therefore, sperm had therefore become significantly larger in males exposed to selection from high levels of sperm competition (Fig. 2A–C). By contrast with sperm length, we found no divergence in testis size (or body size) following histories of selection under intense versus relaxed sperm competition (Fig. 2D), indicating that males under both regimes made similar overall levels of investment to spermatogenesis, possibly as a result of equal and parallel selection from sperm competition and mating frequency, respectively (e.g., Reuter et al. 2008; Crudgington et al. 2009).

Given similar investment into spermatogenesis, the divergence in sperm competitiveness and sperm length between our selection regimes indicates that more intense sperm competition can select for qualitative improvements within individual sperm cell phenotypes, and not necessarily a basic drive to increase sperm numbers. Longer sperm may achieve greater mobility or velocity (Lüpold et al. 2009; Fitzpatrick et al. 2010), providing a competitive advantage if races for fertilization are important (Gage et al. 2004). However, despite the seemingly intuitive relationship between flagellum length and speed, evidence linking the two is ambiguous (Simpson et al. 2014; reviewed in Snook 2005), and may depend upon physical complexities that affect hydrodynamics of very small flagellated cells (Humphries et al. 2008). Notably, in experiments with externally fertilizing myobatrachid frogs, it is slower swimming (potentially longer lived) sperm that win more competitive fertilizations (Dziminski et al. 2009) and, across myobatrachid species and Chinese anurans, there are significant positive relationships between level of sperm competition and sperm length (Byrne et al. 2003; Zeng et al. 2014). Increasing flagellum length will theoretically deliver additional mechanical thrust (Katz and Drobnis 1990), but that may be translated into different swimming patterns. For example, thrust and torque, not speed, may be advantageous in species where females store sperm from multiple males at high densities in narrow tract tubules, with selection on sperm to resist displacement and secure optimal storage sites for fertilization (Immler et al. 2011; Lüpold et al. 2012). Both bursal and spermathecal storage sites are invariably densely packed with sperm following mating opportunities in *T. castaneum*, with the position in the bursa being important for proximate fertilization success and storage in the spermathecal tubules possibly playing roles over longer oviposition periods (Droge‐Young et al. 2016). Whatever the specific mechanistic advantage that longer sperm have for fertilization success, our findings provide clear evidence that sperm competition can directionally select for increased investment in sperm size, revealing the importance of qualitative aspects of sperm cell phenotypes that are relevant for models explaining the evolution and maintenance of anisogamy (Parker 1982; Snook 2005; Lüpold et al. 2016).

Very few previous studies have examined the response of sperm length to experimental evolution of mating pattern and sperm competition. Studies using *Drosophila, Callosobruchus*, and house mice (38–81, 90, and eight generations, respectively) found no significant responses by sperm length in regimes experiencing enforced monogamy, compared with regimes allowing polyandry (Pitnick et al. 2001; Gay et al. 2009; Firman and Simmons 2010). Also, in a well‐replicated study employing experimental evolution and controlling for effective population sizes in *Drosophila pseudoobscura*, the lengths and component dimensions of both short and long sperm morphs showed no change after more than 40 generations of selection under elevated promiscuity versus monogamy (Crudgington et al. 2009). This lack of response could not be explained by low genetic variability in either sperm or female tract length, both of which show moderate potential to evolve under direct selection (Snook et al. 2010; Moore et al. 2013). However, in *Caenorhabditis* nematodes, which produce amoeboid sperm, LaMunyon and Ward (2002) compared selfing hermaphrodites (where sperm competition is absent) versus sexually crossing lines (where males compete) and found that sperm evolved to be bigger in the context of sperm competition across 60 generations. Finally, in a study across 20 generations, monogamy did not change sperm lengths of *Macrostomum* flatworms, but length of the sperm bristles did elongate in polygamous lines (Janicke et al. 2016).

Building on cross‐species analyses (e.g., Lüpold et al. 2016), evidence for larger sperm advantages in sperm competition have been previously confirmed where natural sperm size variation exists between individual males within a species (reviewed in Snook 2005). In *Caenorhabditis* nematodes and *Rhizoglyphus* bulb mites, which produce amoeboid sperm, males with larger sperm won more fertilizations within sperm competitions (Radwan 1996; LaMunyon and Ward 1998). However, studies using natural variation in flagellated sperm length in other insect, fish, and mammal species found no longer sperm advantages (Gage and Morrow 2003; Simmons et al. 2003; Gage et al. 2004), although in birds, male zebra finches producing longer sperm win more fertilizations under sperm competition (Bennison et al. 2015). *Drosophila* experiments have provided important insights, with experimental evidence for both qualitative and quantitative advantages for sperm length and number in sperm competition in *D. melanogaster* (Miller and Pitnick 2002; Patterini et al. 2006). Importantly, the qualitative advantages achieved by longer sperm in *Drosophila* competitions are known to interact with the structure of the female reproductive tract, such that long‐sperm advantages only become evident when dimensions of the female tract are also enlarged (Miller and Pitnick 2002).

The *Drosophila* studies revealing that sperm qualitative advantages prevail in the context of variation by the female reproductive tract, demonstrate the existence of “cryptic female choice,” where particular sperm quality phenotypes must co‐adapt in males under selection from competition for fertilizations in a particular female‐controlled environment (Miller and Pitnick 2002; Snook 2005). We measured the relative competitiveness of our selection regime males against standard male competitors (carrying the Reindeer marker) for females of the same regime (Fig. 1). Having run sperm competition trials both within and between either selection regime's three lines (Fig. S1), we found no evidence of line coevolution (Fig. S2). However, our 20% differences in overall sperm competitiveness between male‐ and Female‐biased males may also have been influenced by female postcopulatory processes (Miller and Pitnick 2002; Snook 2005). If longer sperm are costly to maintain, then cryptic female choice could logically drive sperm elongation through sexual selection on male condition via a process where sperm phenotypes act as gametic equivalents of the peacocks’ tail. This possibility has been recently confirmed through comparative analyses revealing that sperm size can exaggerate via Fisherian runaway sexual selection, mediated by cryptic female choice (Lüpold et al. 2016). If sperm cells act as postcopulatory signals of male quality under such sexual selection, then the trait must be honest and costly to develop and maintain (Lüpold et al. 2016). Our experiment comparing testis and sperm length development within males reared on protein‐rich versus protein‐poor diets demonstrates that sperm elongation is indeed costly in *T. castaneum* and is dependent on male condition (Fig. 3), allowing sperm size to represent a reliable signal for postcopulatory sexual selection (Lüpold et al. 2016). Previous work in *T. castaneum* has shown that diet restriction also constrains male fertility and sperm competitiveness (Sbilordo et al. 2011).

In addition to demonstrating that sperm competition can exert directional selection on sperm length, our experiments allow us to test directly the hypothesis that sperm competition generates stabilizing selection on sperm size. If sperm competition levels are high, and there is an optimally competitive sperm length phenotype, then selection could stabilize morphological variation more tightly around the optimum (Parker and Begon 1993; Lifjeld et al. 2010). Such stabilization could act at the population level, driving down between‐male variation in sperm size to the population optimum (Morrow and Gage 2001), or within individual males, driving up quality control within spermatogenesis and reducing production errors to maximize the number of optimally competitive sperm produced (Lifjeld et al. 2010). The converse situation may also apply, where very relaxed postcopulatory sexual selection could allow more variant sperm morphology, perhaps exemplified by species showing exceptionally degenerative sperm morphology for their taxa, and also associated with very low levels of sperm competition (van der Horst and Maree 2014; Stewart et al. 2016). A number of comparative studies using passerine birds (Immler et al. 2008; Kleven et al. 2008; Lifjeld et al. 2010; Laskemoen et al. 2013), murine rodents (Varea‐ Sánchez et al. 2014), and social insects (Fitzpatrick and Baer 2011) have found evidence that decreased sperm length variance, both between and within males, exists where sperm competition levels are higher. Building on these findings, Lifjeld et al. (2010) have proposed that sperm length variance itself could represent an objective index of the intensity of sperm competition sustained by a species. However, we found no evidence, following controlled experimental evolution on a single ancestral population across 83 generations of exposure to divergent levels of sperm competition, that increased sperm competition stabilizes and reduces sperm length variation (Fig. 2E). Our findings therefore do not support the universal use of sperm length variance as an indicator of the level of sperm competition in a population (Lifjeld et al. 2010). However, the cross‐species evidence for sperm competition and stabilizing selection on sperm comes from wild systems (Immler et al. 2008; Kleven et al. 2008; Lifjeld et al. 2010; Varea‐ Sánchez et al. 2014), where environmental variation and a greater range of stresses exist. Under natural conditions, developmental stability within spermatogenesis may be more difficult to achieve, making the existence of sperm size variance in the absence of selection from sperm competition the default condition. Our results showing increased sperm length variance in males exposed to dietary stress, compared with low variance in optimal and benign culture conditions support this idea (Fig. 3E), as does previous work in this system showing increased sperm length variance under genetic stress from inbreeding (Michalczyk et al. 2011).

In conclusion, we applied experimental evolution through variation in adult mating pattern to successfully and significantly diverge sperm competitiveness of independent replicate lines. Although testis and body size measures showed that selection did not change male relative investment into spermatogenesis through experimental evolution, we found that exposure to more intense sperm competition regimes caused males to evolve longer sperm, and there are obvious costs to such sperm elongation under diet restriction. Our findings therefore demonstrate positive directional selection from sperm competition on costly sperm size, revealing that postcopulatory sexual selection can generate qualitative selection on sperm form and function.

Editor in Chief: J. Slate

Associate Editor: R. Snook

## Supporting information

Supporting Information

**Figure S1**. Sperm competition crossing design to balance within‐ versus between‐line potential influences on differential fertilization success.

**Figure S2**. Proportion of offspring sired by males from relaxed Female‐biased (blue) versus intense Male‐biased (red) sperm competition regime in sperm competition with a single rival.

Supporting Information

Supporting Information
