# Supplementary figures and images for: Experimental evolution reveals that sperm competition intensity selects for longer, more costly sperm
Source: Evol Lett. 2017 Jun 7;1(2):102–13. doi: 10.1002/evl3.13 (PMC6089504; doi:10.1002/evl3.13)

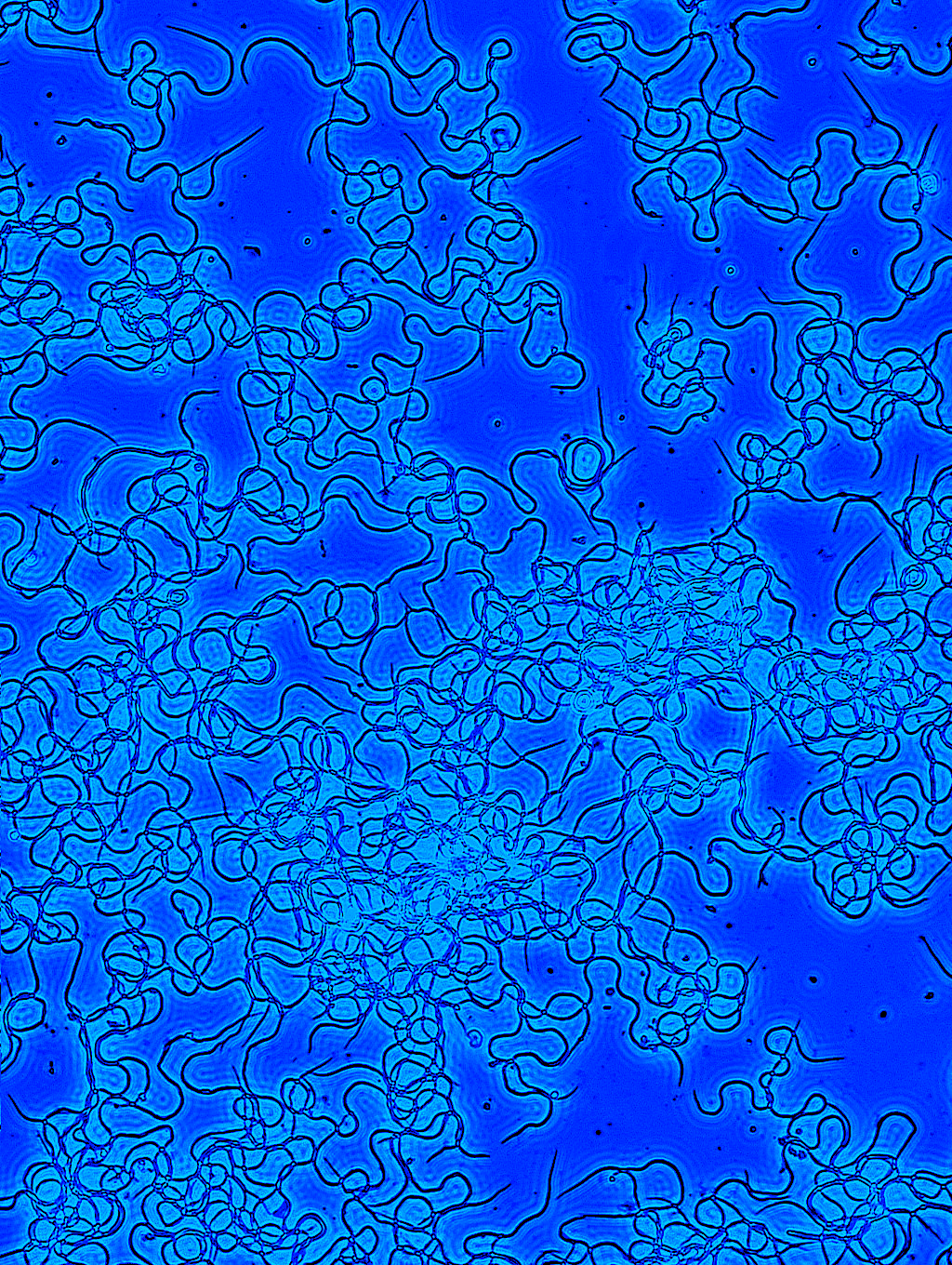

Supplement: Supplementary file 1 — Supporting Information [file EVL3-1-102-s001.tiff]

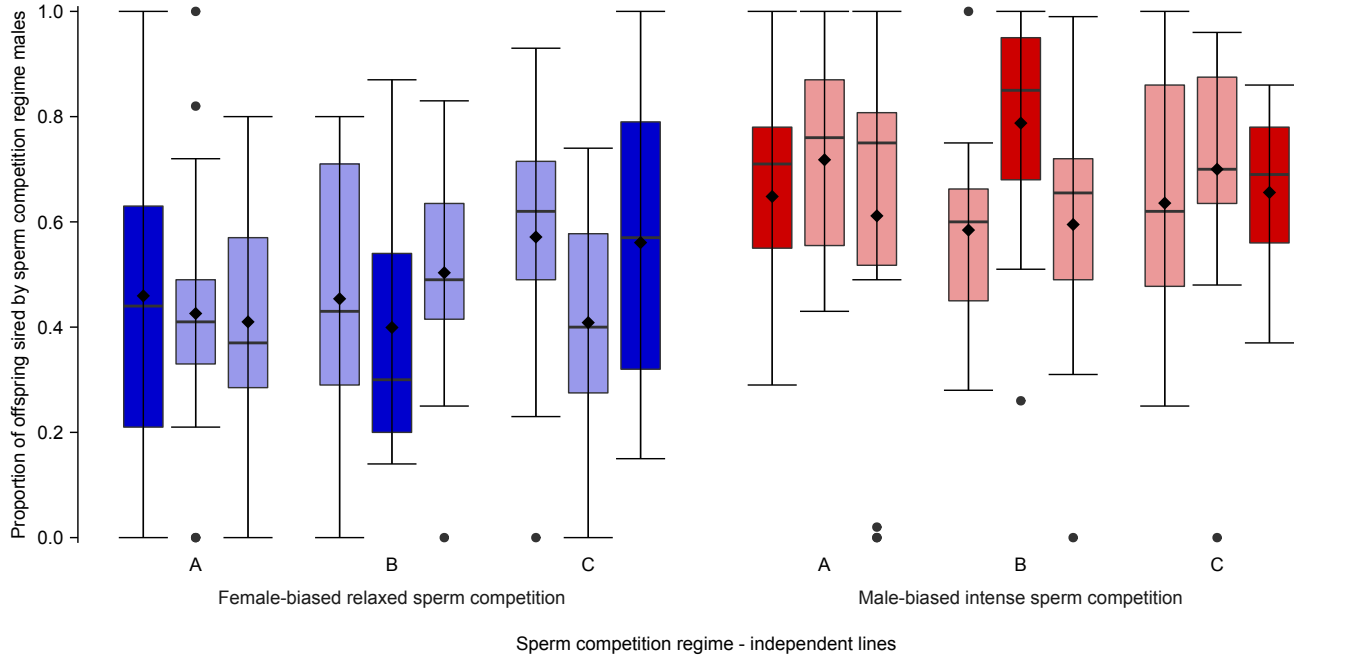

Supplement: Supplementary file 3 — Figure S2. Proportion of offspring sired by males from relaxed Female‐biased (blue) versus intense Male‐biased (red) sperm competition regime in sperm competition with a single rival. [file EVL3-1-102-s003.pdf]

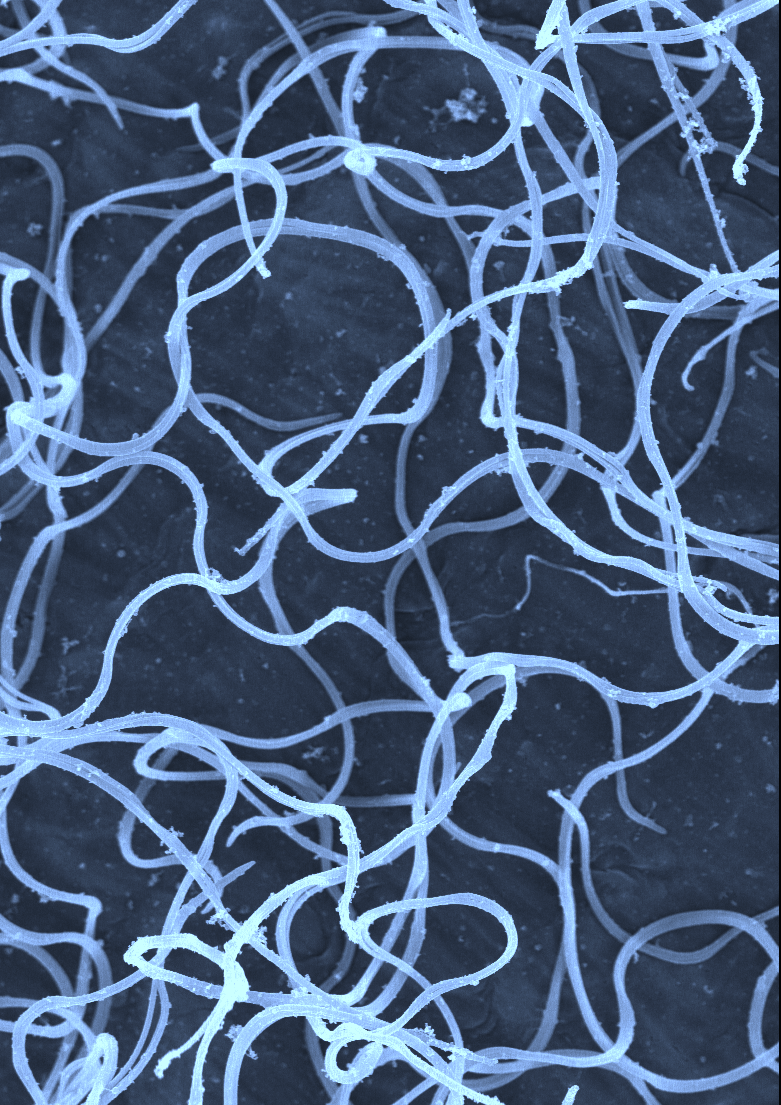

Supplement: Supplementary file 4 — Supporting Information [file EVL3-1-102-s004.tiff]

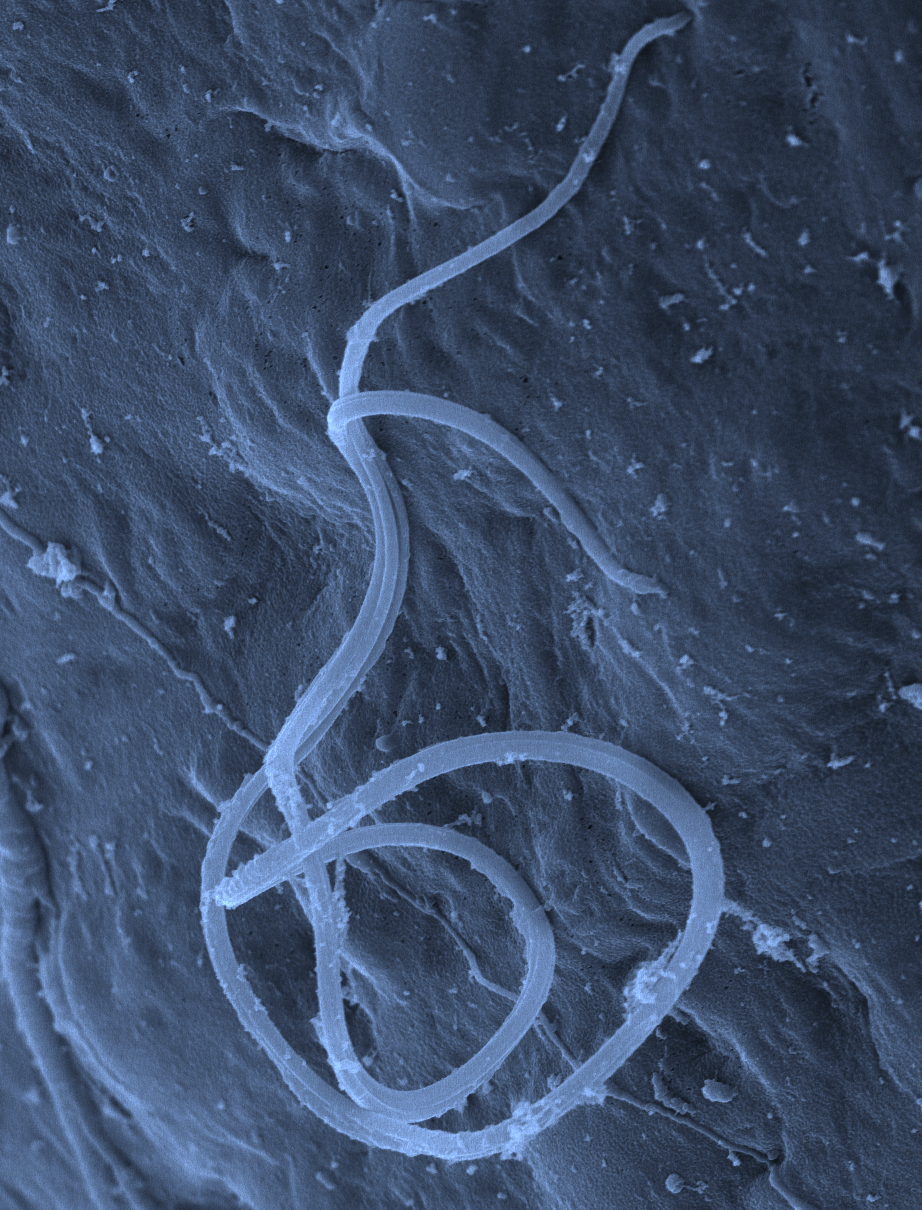

Supplement: Supplementary file 5 — Supporting Information [file EVL3-1-102-s005.tiff]
