# Supplementary material for: Experimental evolution reveals that sperm competition intensity selects for longer, more costly sperm
Source: Evol Lett. 2017 Jun 7;1(2):102–13. doi: 10.1002/evl3.13 (PMC6089504; doi:10.1002/evl3.13)
Supplement: Supplementary file 2 — Figure S1. Sperm competition crossing design to balance within‐ versus between‐line potential influences on differential fertilization success. [file EVL3-1-102-s002.pdf]

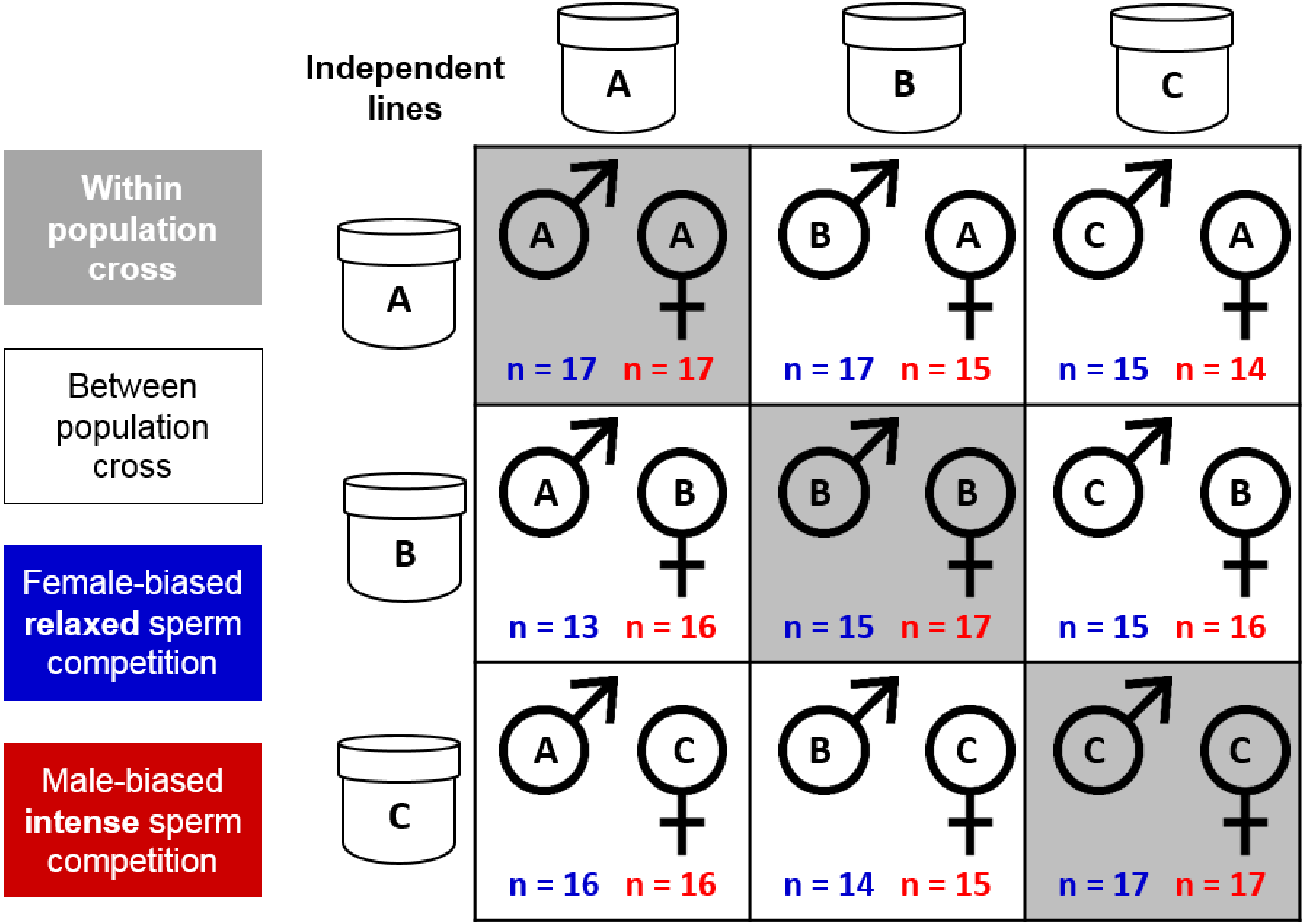
Independent lines
A
B
C
Within population cross
Between population cross
Female-biased relaxed sperm competition
Male-biased intense sperm competition
A
B
C
A ♂ A ♀ n = 17 n = 17
B ♂ A ♀ n = 17 n = 15
C ♂ A ♀ n = 15 n = 14
A ♂ B ♀ n = 13 n = 16
B ♂ B ♀ n = 15 n = 17
C ♂ B ♀ n = 15 n = 16
A ♂ C ♀ n = 16 n = 16
B ♂ C ♀ n = 14 n = 15
C ♂ C ♀ n = 17 n = 17
